# Progress in Pediatric Asthma Surveillance I: The Application of Health Care Use Data in Alameda County, California

**Published:** 2006-06-15

**Authors:** Paul B English, Eric M Roberts, Stephen K Van den Eeden, G. Thomas Ray

**Affiliations:** California Department of Health Services, Environmental Health Investigations Branch; California Department of Health Services, Environmental Health Investigations Branch; Kaiser Permanente of Northern California, Division of Research, Oakland, Calif; Kaiser Permanente of Northern California, Division of Research, Oakland, Calif

## Abstract

**Introduction:**

The ability to conduct community-level asthma surveillance is increasingly crucial for public health programming and child health advocacy. We explored the potential and limitations of health care use records from both public and private sources for asthma surveillance in a California county.

**Methods:**

We combined administrative patient record data from Kaiser Permanente of Northern California and Medi-Cal (the California Medicaid program) for Alameda County residents during 2001. We assessed the resulting data set for completeness, population representation, consistency with external data, and internal indicator consistency.

**Results:**

Our resulting data set included records for 226,383 children younger than 18 years. Completeness of Medicaid data was affected by managed care market share, reducing our usable data set size to 176,789, approximately equal to one of every two children in the county or one of every 3 person-months. External data documenting hospitalization rates due to asthma were poorly correlated with hospitalization rates (*r* = 0.2120, *P* = .20) but highly correlated with emergency department visits (*r* = 0.8607, *P* <.001) in the resulting data set. High internal consistency of indicators suggested that the data set represented a broad spectrum of health care access and quality of care congruent with clinical aspects of the disease.

**Conclusion:**

The utility of these data is affected by logistical and administrative factors, including the health care payment structure and the market shares of care providers. These factors can be expected to similarly affect the utility of this approach in other counties. Our ability to generate county-level health statistics for comparison with other locations was limited, although the data set appeared well suited for within-county geographic analysis. In light of these findings, these data have the potential to expand the local health surveillance capacity of communities.

## Introduction

Although asthma has emerged as a major public health challenge (1), our current needs for asthma surveillance have far outpaced our capabilities. This problem comes as no surprise because there is no single test or entirely objective definition for either the disease or its resolution (2-4). Historically, surveys of patients and their parents have been the backbone of asthma surveillance. Cultural differences and inconsistencies in reporting (5-8), however, as well as the high cost of survey studies (2,9) make such methods problematic for ongoing monitoring. At the national level, asthma surveillance takes the form of a patchwork of surveys that report symptoms, diagnoses, emergency department and clinic visits, and hospitalizations (1,10). A few states have also taken steps toward asthma surveillance (11-13). During the past several years, a consistent picture has emerged of generally rising morbidity and stark social disparities of the disease, but the causes of both the spread and the disparities remain controversial.

Increasingly, childhood asthma prevention and management have become a state and local issue as state health care expenditures have increased and communities have focused on the impacts of the disease on school districts, local economic development, and questions of environmental justice (14-17). Communities seek local asthma surveillance data to enable them to assess small-area variations in the burden of disease, identify subpopulations at risk, and plan health resource allocation; however, such data are generally scarce (18,19). As asthma is increasingly recognized as being tied to issues of neighborhood segregation, local air quality, and distribution of health resources, the demand for local surveillance of the disease can be expected to increase.

As part of the California Environmental Public Health Tracking Program (CEHTP), funded by the Centers for Disease Control and Prevention (CDC), we collaborated with a private health care provider, Kaiser Permanente of Northern California; Medi-Cal, California's Medicaid program; and an array of community-based and nongovernmental organizations to develop asthma surveillance that would meet the needs of stakeholders in Alameda County, a mostly urban county in the metropolitan San Francisco Bay area. We were interested in our ability to generate high-quality data that 1) represented the county population, 2) provided a complete picture of the geography of asthma using various asthma-related health events, and 3) included patient home addresses to enable high-resolution geographic analysis. 

The use of health care services claims for monitoring asthma morbidity, health care access, and management has been emphasized as an important next step (20,21), although few (13,22-24) have described in detail the possibilities, pitfalls, and limitations of such work. In this article, we describe our process of evaluating the utility of health care use data for asthma surveillance and discuss the logistical and administrative factors affecting data utility. Technical and statistical procedures for data analysis, visualization, and surveillance findings are described in a companion article (25) in this issue of *Preventing Chronic Disease*.

### Health care use records for asthma surveillance

Analysis of hospitalization rates to reflect the impact of asthma in populations is a long-standing practice (26), although it has several limitations. Among children, only those with asthma that is severe, poorly controlled, or both are hospitalized, which means that hospitalization rates are confounded by differences in access to care among populations (10,27,28). Furthermore, hospitalization is a comparatively rare event relative to overall disease prevalence, so small-area rates are usually difficult to calculate with any precision. Some epidemiologists have used 3-year averages to gain statistical stability at the postal ZIP-code level, but this seems to be the limit of geographic resolution for hospitalization data (29). Oyana et al (30) were able to analyze geographic clustering of asthma hospitalizations around the Peace Bridge complex in Buffalo, NY, by aggregating 5 years of such data.

**Figure 1:**
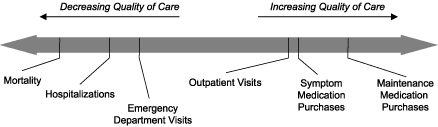
The spectrum of health care use indicators for asthma by quality of care.

Several investigators have explored the use of health care billing records for asthma surveillance, an approach that offers several advantages. Interest has centered on the possibility that health care events other than hospitalization, such as emergency department visits, outpatient visits, and symptom and maintenance medication purchases, may be available for surveillance purposes, which could greatly expand the pool of people identified as having asthma. In populations with good access to care, mortality, hospitalizations, and emergency department visits would not be expected to be elevated even with a relatively high prevalence of asthma (31) (Figure 1). Similarly, among populations with poor access to care, these indicators would be expected to be elevated out of proportion to the asthma prevalence. Our hope is that assessing the rates of lower acuity or routine events will lead to a more complete picture of asthma in geographically defined populations.

Some efforts have been made to evaluate the usefulness of billing records for asthma surveillance in Canada (32). A few studies have also used insurance claims data for the study of occupational disease (33-35). A group of investigators in Milwaukee (23) sought out records from area hospitals, clinics, and health maintenance organizations (HMOs) to estimate asthma burden. They were able to monitor both emergency department and outpatient visits (in addition to hospitalizations) for asthma in a relatively low-cost, potentially sustainable manner.

Other researchers have attempted to use medication purchasing patterns to construct indices of asthma in populations. This approach is consistent with U.S. national guidelines, which explicitly include medication use when classifying asthma severity (31). A Canadian group was able to roughly classify patients according to the severity of their asthma using medication records (24), although it cautions that such an approach in the United States would be confounded by differences in health care access among populations. A North Carolina group examined health care use, including medication purchasing, among Medicaid beneficiaries and was successful in quantifying prevalence by age, race, and rural or urban residence (36). A consensus is emerging that medication purchasing information is an important component of records-based asthma surveillance that requires development (10,26).

All of these studies have cited the need for comprehensive indicators of asthma impact beyond hospitalization rates as a motivation for investigating the use of health care billing records. Curiously, none mentions an additional advantage related to the use of such records — the availability of patient address data to enable surveillance of asthma in small geographic areas. Advances in geographic coding, spatial statistics, and geographic information mapping have made possible the calculation and presentation of disease rates at sub–ZIP-code resolution while preserving patient confidentiality (37).

### Evaluating health care use records for surveillance purposes

We evaluated the utility of health care use records from public and private sources for monitoring asthma among children. We were particularly interested in the feasibility of using these data to meet the surveillance needs of local stakeholders, which included quantifying and visualizing health disparities, identifying populations at elevated risk for asthma, and informing discussions of environmental justice by linking them to broader population health issues.

To evaluate this feasibility, we constructed a working data set for Alameda County, a diverse, urban county in Northern California of 1.4 million residents. We examined 1) data completeness, 2) resulting population representation, 3) external consistency of the data with the previously understood distribution of asthma in Alameda County, and 4) internal consistency of the indicators with each other.

## Methods

### Data sources

Health care use data were drawn from two sources. The first was Kaiser Permanente of Northern California (KPNC), an integrated health care delivery system that is the region's largest single provider of health services. Out of the total 3.1 million members of KPNC, 577,687 were residents of Alameda County during 2001; approximately 40% of county residents received their care at KPNC during that year. Kaiser Permanente maintains a complete list of enrollees (denominator data) and databases describing hospitalizations, clinic visits, referrals, external claims, and medication purchases by members (numerator data) and uses these for both administrative and health care services research purposes. (For examples, see Davis et al [38] and Schoen et al [39].)

**Figure 2:**
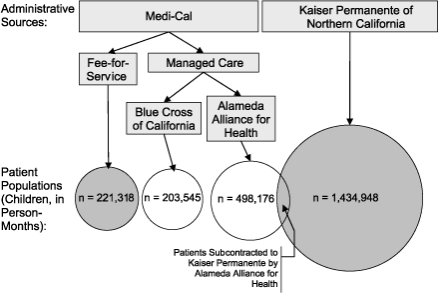
Administrative sources for health care billing records in Alameda County, California. Shaded circles indicate enrollee populations able to be included in the analysis because of completeness of records. Approximately 3.9% of Kaiser Permanente person-months represent Medi-Cal beneficiaries subcontracted through Alameda Alliance for Health.

The second source was Medi-Cal, which covered 227,086 beneficiaries in Alameda County during 2001. Medi-Cal is a complicated data source because the state has subcontracted patient care through HMOs since 2001. As of 2001 in Alameda County, all patients qualifying for zero share-of-cost Medicaid benefits were required to enroll in one of the two managed care plans in the county, Blue Cross of California or Alameda Alliance for Health. (Other beneficiaries are given the option to join.) We investigated the reporting rates of health care use by the HMOs to the state Department of Health Services in Sacramento by comparing them to use rates under the fee-for-service arrangement. Finally, a small portion of Kaiser Permanente enrollees (1.7% of the total, 3.2% of children) are also Medi-Cal patients subcontracted through the Alameda Alliance for Health (Figure 2).

We compared these data to hospitalization counts abstracted by the California Office of Statewide Healthcare Planning and Development (OSHPD). Hospitals are required by law to report these data to OSHPD; the data represent 100% of hospitalizations in the state. Although hospitalizations are the only asthma-related event recorded by OSHPD, and the patient's ZIP code of residence is the sole geographic field available, these data are the nearest thing to a gold standard to which we can compare numbers generated from the Kaiser Permanente and Medi-Cal data sets.

### Defining asthma events

Hospitalizations, emergency department visits, and outpatient visits in the data sets included fields for primary and, in certain cases, secondary diagnoses. For comparisons with existing data sets, such events were considered asthma-related if the primary diagnosis began with the digits *493*, the *International Classification of Diseases, Ninth Revision, Clinical Modification* (*ICD-9-CM)* code for asthma. For health surveillance, however, we also included events with asthma as the secondary diagnosis and a primary diagnosis of *pneumonia*, *respiratory failure*, or another condition for which an asthma exacerbation was likely to be the cause.

We classified medications as asthma related based on their functional class and divided them into *symptom* (or *rescue*) and *maintenance* (or *controller*) medications. Symptom medications included long- and short-acting beta agonists and anticholinergics; maintenance medications included antileukotrienes, mast cell stabilizers, methylxanthines, and inhaled corticosteroids. (The corticosteroids could be present alone or in combination with bronchodilating agents). Oral corticosteroids were omitted because of concern that their use for reasons other than asthma was commonplace enough to compromise the specificity of the indicator. The classification of long-acting beta agonists as symptom rather than maintenance medications was subject to extensive debate. In the end, the lack of evidence (40) for long-term efficacy of these drugs, particularly in preventing hospitalizations, argued against their classification in the maintenance category. It was later noted that this classification algorithm is identical to that constructed independently by Buescher and Jones-Vessey (36). 

### Patient addresses

Home addresses were obtained from records directly maintained by KPNC and Medi-Cal. These were standardized using ZP4 (Semaphore Corp, Pismo Beach, Calif); they were subsequently geocoded using a custom application written in Java 2 Platform, Standard Edition (Sun Microsystems Inc, Santa Clara, Calif) and ArcSDE version 9.0 (ESRI, Redlands, Calif). Geocoded address coordinates were taken from the first successful match of the following four street centerline data sets (in order): Dynamap/2000 version 13 (Tele Atlas, Lebanon, NH), Navstreets (Navteq, Chicago, Ill), Tele Atlas MultiNet (Tele Atlas, Lebanon, NH), and the Census 2000 TIGER/Line (U.S. Census Bureau, Washington, DC). For each street centerline data set, the first attempt to match an address was made by indexing the address's ZIP code. Failing that, the soundex phonetic code of the address's city was matched against an index of the soundex phonetic code of the street centerline's post office name, based on its ZIP code. We have noted the spatial accuracy of assignment to census tracts by this method to be approximately 99%.

### Statistical analysis

To measure data completeness, we used a straightforward frequency analysis. For assessment of population representation, we used frequency analysis preceded by the coding of each patient to the census tract of residence, roughly following the methods of Krieger (41). To determine the consistency of indicators with external data sources and among each other, we applied bivariate correlation analysis to data aggregated to the ZIP-code and census-tract levels, respectively. For the ZIP-code level, we calculated the Pearson correlation coefficients between the OSHPD ZIP-code–level hospitalization rates and the Kaiser Permanente hospitalization rates, the Medi-Cal fee-for-service rates, both combined, and the emergency department visit rates from both combined. For all analyses, we used SAS version 8.02 (SAS Institute, Cary, NC). 

## Results

The Medi-Cal fee-for-service and KPNC data combined to represent 226,383 children younger than 18 years, or 2.3 million person-months of information. Usable sample size was subsequently reduced in light of data quality considerations.

### Data completeness

**Figure 3:**
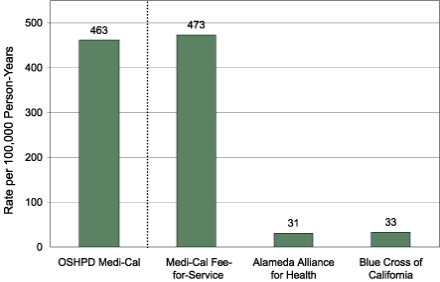
Hospitalization rates attributable to asthma (primary diagnosis only) among Medi-Cal beneficiaries during 2001 in Alameda County, California, by source of data. The Office of Statewide Healthcare Planning and Development (OSHPD) includes 100% of county residents receiving Medi-Cal benefits; the other three data sources include only their respective portions of this population. The OSHPD rate is calculated for children aged 0 to 14 years; all other rates are for children aged 0 to 17 years.

The hospitalization rate calculated for Medi-Cal fee-for-service seems to be consistent with the rate calculated using the OSHPD data (Figure 3). Both managed care subcontractors in the county, however, reported hospitalizations at a substantially lower rate than either of these sources. Managed care rates of emergency department visits, outpatient visits, and medication purchases also consistently amounted to a small fraction of those calculated using Medi-Cal. For this reason, subsequent analyses excluded the managed care Medi-Cal population, reducing the total sample size to 176,789 children, or 1,656,266 person-months.

**Table:** 

### Population representation

**Figure 4:**
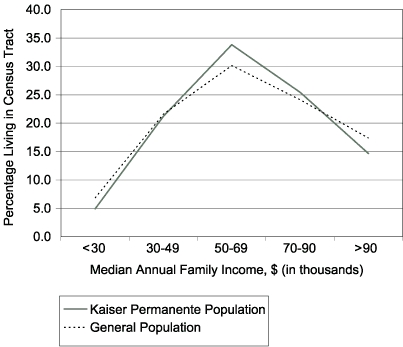
This graph shows the distribution of the Kaiser Permanente of Northern California enrollee population by category of annual family income. The y axis is labeled “Percentage Living in Census Tract” and the x axis is labeled “Median Annual Family Income” divided into five categories: less than $30,000; $30,000–$49,000; $50,000–$69,000; $70,000–$90,000; and more than $90,000. The graph shows approximately 5% of the enrollee population in the less-than-$30,000 category; the graph peaks at approximately 34% in the $50,000–$69,000 category and decreases to approximately 15% in the more-than-$90,000 category. The figure also shows the income distribution of the general population for the county, which shows a similar pattern. Approximately 7% of the general population are in the less-than-$30,000 category; the graph peaks at approximately 30% in the $50,000–$69,000 category and decreases to approximately 17% in the more-than-$90,000 category. The enrollee population shows high congruence with the general population of the county, although it slightly underrepresents populations in the lowest and highest income categories and overrepresents those in the middle category.

Comparison with fee-for-service beneficiaries revealed that managed care enrollees were more likely to be younger than 18 years (61% compared with 21%), and among children, they were more likely to be enrolled in Medi-Cal for the entire 12 months of the year (55% compared with 19%). For Kaiser Permanente data, we coded enrollees to their census tract of residence. In this manner, we could estimate the proportion of the sample in each income stratum and compare these figures to those for the overall population using 2000 U.S. census data. The enrollee population shows high congruence with the overall socioeconomic profile of the county, although it slightly underrepresents populations on either extreme of the continuum and overrepresents those in the middle (Figure 4).

### Consistency with external data

We assessed the validity of our data by comparing them with the OSHPD data set, from which hospitalization rates due to asthma were available by ZIP code aggregated from 1998 to 2000. Neither data set individually nor combined produced a picture of the geographic distribution of asthma hospitalizations consistent with the external data set (Table 1). Among the county's health care providers, Kaiser Permanente hospitalization rates tend to be low (approximately 50% of the countywide rate in 2001); this reduction seems to apply throughout the county, because the variance across ZIP codes is much lower for KPNC enrollees (78.33) than it is for the population as a whole (1499.33). Given the large number of KPNC enrollees in the data set, the low correlation of hospitalization rates is likely to be because of this decrease in variance.

Rates of emergency department visits for the two data sets, however, were much more highly correlated with OSHPD asthma hospitalizations, and this correlation was higher for both of the data sets combined (*r*  = 0.8607, *P* < .001) than it was for either one alone. Thus, ZIP codes with high OSHPD asthma hospitalization rates also tended to have high emergency department visit rates as contained in our data sets, which is consistent with the notion that people in these areas may have more severe asthma, poorly controlled asthma, or both.

### Internal consistency

We posited that the quality-of-care profile of each census tract within the county would fall along a spectrum of access and quality of care similar to that shown in Figure 1. Pearson correlation coefficients between the health care use indicators at the census tract level are shown in Table 2. The indicators closest to each other on the spectrum of care (such as hospitalizations and emergency department visits) in Figure 1 are indeed the most highly correlated, whereas those farther apart (such as hospitalizations and maintenance medication purchases) are progressively less correlated with each other.

After removal of incomplete records, the data set described a total of 311,774 asthma-related health care events among 1,656,266 person-months, not including hospitalizations. The numbers of each event type are shown in Table 3.

## Discussion

In this study, we evaluated the use of routinely collected health care use data that could be used for an ongoing asthma surveillance system in California. By focusing on administrative health care use data from private and public sources, we were able to make available a range of asthma indicators much broader than our previous hospitalization data alone. The use of these databases also provides an additional advantage for surveillance because patient data are available at the home-address level. We assessed the quality of the assembled data for completeness, population representation, consistency with external sources describing the distribution of asthma morbidity in the county, and internal consistency of the indicators with each other.

### Data completeness

We knew at the outset that data completeness would be most open to questions concerning the state (Medicaid) data, and analysis showed that the key determinant of completeness was the extent to which the beneficiary population was enrolled through managed care rather than fee-for-service systems. For Alameda County in 2001, fee-for-service enrollees had complete data through the state office in Sacramento, whereas managed care enrollees did not. This finding has important implications for the development of asthma surveillance systems using Medicaid data, because 48 of the 53 U.S. states and territories with Medicaid programs use capitated managed care plans, with enrollment ranging from less than 10% to up to 100% of the Medicaid population (42). The total proportion of Medicaid enrollees served through managed care plans increased from 40% in 1996 to more than 60% in 2003 (42), and although some states have a uniform set of managed care options for all enrollees statewide, some (including California) vary by county (42). Under these arrangements, routine reporting of health care events and centralization by the state program is more likely to be incomplete or absent than under traditional fee-for-service systems.

### Population representation

Medicaid data provide a picture of health care use for the county's lowest-income residents; our interest in working with Kaiser Permanente data, in contrast, stemmed from the possibility of representing health care use by people from various socioeconomic strata. Consistent with the findings of Krieger in 1992 (41), we noted that the Kaiser Permanente data performed this role well, although they slightly underrepresented individuals at either extreme of the spectrum and slightly overrepresented those in the middle. It is unclear to what extent the underrepresentation of these extremes may affect asthma surveillance; it is hoped that the inclusion of Medi-Cal enrollees may offset the underrepresentation of low-income communities. As with our findings on the completeness of Medicaid managed care data, these findings should be expected to vary by county in California because multiple managed care entities control varying portions of the state health care market by region.

Our ability to assess the degree to which the fee-for-service portion of the Medi-Cal population was representative of the entire Medi-Cal population was limited. Racial and ethnic data recorded by Medi-Cal, when collected, are considered unreliable, although we could discern that managed care beneficiaries were more likely to be children, and among children they were more likely to be longer-term beneficiaries. The process by which Medi-Cal beneficiaries are assigned to managed care or fee-for-service plans is complex, with choice of assignment contingent upon the administrative mechanism of eligibility. This finding suggests that episodic enrollment is more common among fee-for-service beneficiaries and that enrollment in managed care plans increases with the duration of eligibility.

Overall, the nonrandom nature of the study sample is a limitation; it precludes the calculation of countywide asthma-related health care use rates for comparison to state or national figures. On the other hand, one of every two child residents of the county is included in the data set, or one in three if the figure is calculated using person-months. Because of this fact and the finding that the data set had high external validity, we felt that the data set was useful for within-county sociodemographic and geographic comparisons.

### External validity

Our ability to mirror the geographic patterns known from the OSHPD gold standard data set using the health care event data sets could not be taken for granted. On one hand, we had a private managed care data set with high overall population representation but with a population for whom hospitalization rates (but not emergency department visits) were known to be lower than the countywide average. On the other hand, we had a public payer data set restricted to the communities with the lowest incomes in the county — communities also known to experience a disproportionate share of hospitalizations due to asthma. It was not clear whether one of these data sets, or both data sets combined, would most accurately reflect the overall geographic distribution of asthma events.

Neither the patient records for Medi-Cal, Kaiser Permanente, nor both combined generated a geographic pattern of hospitalization rates particularly consistent with the OSHPD data. We believe this failure to be attributable to the low hospitalization rates across the county for KPNC enrollees, which compresses the variance of this variable. Rates of emergency department visits, by contrast, correlated highly with OSHPD hospitalizations, particularly for the combined data set. Part of the advantage of the combined data set may originate from superior representation across ZIP codes of socioeconomic strata since KPNC underrepresents families with very low incomes, who are disproportionately represented among individuals hospitalized for asthma. Medi-Cal, by contrast, overrepresents those with low incomes but has beneficiaries in fewer ZIP codes around the county. The combined data set may capitalize on the strengths of each. Finally, among children aged 0 to 14 years, the age distribution of the combined data set more closely matches the general population than either one alone (data not shown).

Emergency department visits were highly correlated with hospitalization rates from OSHPD data even though hospitalization rates and emergency department visit rates, being separate phenomena, might not be expected to match each other precisely. Because both hospitalizations and emergency department visits can be expected to reflect the distribution of severe, poorly controlled asthma in the county, however, it is logical that the geographic variations of each should correlate somewhat. Furthermore, Kaiser Permanente, as a centrally coordinated managed care organization, is able to steer patients away from hospital admissions by encouraging extensive emergency department management before making decisions to admit or discharge patients and implementing case management strategies in the interests of both cost savings and quality of care. Because our hospitalization rates are numerically driven by the Kaiser Permanente patients, it makes sense that our hospitalization rates, but not our emergency department visit rates, would be depressed in both magnitude and variance relative to the countywide numbers from OSHPD. Therefore, for analytical purposes (25), we focused on emergency department visits as our indicator of severe asthma, poorly controlled asthma, or both and excluded hospitalizations.

Countywide, rates of other asthma-related health care use were largely consistent with national data. Emergency department visit rates based on the National Ambulatory Medical Care Survey (NAMCS) (1) for this age group are very close to the rates found in this study population. Medication purchasing rates reported from the Medical Expenditure Panel Survey (43) are also consistent with this population, although the Alameda County population seems to purchase a greater proportion of maintenance medications than would be expected from the national numbers. Outpatient visit rates were also substantially higher in the Alameda County population than would be expected based on the national NAMCS numbers (1). Considering the large numbers of KPNC enrollees in the data set, both of these differences may be because of asthma management strategies promoting primary and preventive care within that organization.

### Internal validity

The high degree of internal consistency of the health care use indicators noted in this data set reinforced our view of these indicators as representing various positions on a spectrum of asthma quality of care (Figure 1). Events that would be expected to occur together (such as hospitalizations with emergency department visits or symptom medication purchases with maintenance medication purchases) indeed did so, and events associated with different standards of asthma primary care (such as hospitalizations and maintenance medication purchasing) did not. The ability to reflect this spectrum of quality of care was a major objective for developing these data sets because of stakeholder interest in comprehensively representing the problem of asthma in the county.

### Other measures of data utility

Other authors have also discussed attributes for the assessment of data sources for health surveillance purposes (44). These include quantitative attributes such as sensitivity (completeness), representativeness, validity, and timeliness, as well as qualitative attributes such as simplicity, acceptability, and cost. We directly assessed all of the qualitative attributes in this study except for timeliness. Medi-Cal patient encounter data have taken an average of 4 months for processing before becoming centrally available through the Medical Care Statistics Section of the California Department of Health Services, although the speed of this process appears to be increasing. One problem with receiving data is the time needed to screen it for quality assurance purposes. Sacrifices in data quality may be necessary to increase the value of the data for surveillance with the understanding that surveillance data results are preliminary. Similarly, it is the experience of KPNC's Division of Research that a lead time of approximately 6 months is required before complete patient encounter data are available. This provider is currently developing a comprehensive electronic medical record system, however, which is expected to make data available almost in real-time.

### External factors affecting data quality

The potential to adapt the data sources for surveillance, particularly outside of the demonstration area of Alameda County, is affected by several issues. A primary requirement for this approach to be adopted elsewhere would be the ability to incorporate private sources of data that represent a large market share of health services coverage in that location. Similarly, the proportion of Medi-Cal data that is fee-for-service or managed care varies by county. We expect that these two issues will play the greatest role in determining the extent to which similar systems could be implemented in other localities in the state or nationally.

Automated geocoding and address verification systems that were developed for this project could be used in other counties to make the analytic process more efficient. Similarly, the adoption of data standards by all managed care entities would be of great value for surveillance. With collaborations among private local providers of health care use data, data costs could be minimal. However, there are additional costs for data processing, data visualization and analysis, hardware, software, personnel, and data dissemination.

Finally, in contrast to concerns expressed by many colleagues, the provisions of the Health Insurance Portability and Accountability Act of 1996 (HIPAA) did not constitute an obstacle for the conduct of this work. The fundamentally research-oriented objectives of the project partly explain this lack of difficulty, as did the absence of intent to contact any of the patients described in the database. Safeguards for the protection of confidential information were prepared in advance, and the use of density estimation mapping for the visualization of the data (25) was seen as a further protection of confidentiality. Full authorization from the state Department of Health Services Committee for the Protection of Human Subjects was sought and obtained before the project began.

We constructed this working data set to increase asthma surveillance capacity to facilitate clinical and public health interventions. For two reasons, the result represents a substantial step forward. The first is the fact that we were able to incorporate a range of asthma-related indicator variables into a single data set with ample statistical power (Table 3), meeting a need expressed by several researchers (10,24,26). Furthermore, because of the availability of patient address data, we enable high-resolution geographic analysis of asthma in the county, as demonstrated in our companion article (25). Through this activity, we are able to identify subpopulations facing increased vulnerability to asthma and barriers to care, quantify socioeconomic disparities in patterns of asthma care, and formulate hypotheses about local sources of pollution or other environmental contributors to the impact of disease.

## Figures and Tables

**Table 1:** Correlations of Asthma Hospitalization and Emergency Department (ED) Visit Rates for Children Aged 0 to 14 Years From 2001 Surveillance Data Sets With Countywide Childhood Asthma Hospitalizations^a^, Alameda County, California

**Health Care Event**	**No. of ZIP Codes^b^**	**r**	***P* Value**
**Hospitalizations**			
Kaiser Permanente only	31	0.2625	.15
Medi-Cal fee-for-service only	14	−0.2763	.34
Both	38	0.2120	.20
**ED visits**			
Kaiser Permanente only	36	0.7649	<.001
Medi-Cal fee-for-service only	17	0.2784	.28
Both	38	0.8607	<.001

a Office of Statewide Healthcare Planning and Development 100% hospitalization data set, children aged 0 to 14 years, 1998 to 2000.

b To minimize the influence of unstable rates on correlation coefficients, only postal ZIP codes with four or more ED visits were included in calculations.

**Table 2:** Internal Consistency of Asthma-related Health Care Use Indicators

	**Hospitalizations**	**ED Visits**	**Outpatient Visits**	**Symptom Medication Purchases**	**Maintenance Medication Purchases**

	**r *P * No. of Census Tracts**	**r *P * No. of Census Tracts**	**r *P *No. of Census Tracts**	**r *P *No. of Census Tracts**	**r *P *No. of Census Tracts**
**Hospitalizations**	1.0000NA262	0.5980<.001262	0.2331<.001232	0.0893.15262	0.0049.94262
**ED visits**	—	1.0000NA302	0.4072<.001302	0.2213<.001302	?0.0266.64302
**Outpatient visits**	—	—	1.0000NA321	0.5197<.001302	0.4061<.001321
**Symptom medication purchases**	—	—	—	1.0000NA321	0.7448<.001321
**Maintenance medication purchases**	—	—	—	—	1.0000NA321

ED indicates emergency department; NA, not applicable.

**Table 3:** Asthma-related Health Care Events Available for Health Surveillance From Combined Data Set (n = 176,789 Children Aged 0 to 17 Years, or 1,656,266 Person-Months)

**Health Care Event**	**Total**
Hospitalizations	0^a^
Emergency department visits	3,579
Outpatient visits	53,611
Purchases of symptom medications	160,029
Purchases of maintenance medications	94,555

a Not used because of poor external validity.
